# The effect of a course of selected corrective exercises on posture, scapula-humeral rhythm and performance of adolescent volleyball players with upper cross syndrome

**DOI:** 10.1186/s12891-023-06592-7

**Published:** 2023-06-14

**Authors:** Morteza Homayounnia Firouzjah, Ebrahim Mohammad Ali Nasab Firouzjah, Zahra Ebrahimi

**Affiliations:** 1 Department of Physical Education, Farhangian University, P.O. Box 14665-889, Tehran, Iran; 2 Department of Exercise Physiology and Corrective Exercise, Faculty of Sport Sciences, Urmia University, Urmia, Iran; 3 Department of Exercise Physiology and Corrective Exercise, Faculty of Sport Sciences, Urmia University, Urmia, Iran

**Keywords:** Abnormality, Corrective Exercise, Sports performance, Volleyball

## Abstract

**Background:**

This study aims to investigate the effect of a course of selected corrective exercises on posture, scapula-humeral rhythm and performance of adolescent volleyball players.

**Methods:**

30 adolescent volleyball players with upper cross syndrome were purposefully selected and assigned into 2 control and training groups. The degree of back curvature was evaluated using a flexible ruler, forward head and forward shoulder size by photographic method, scapula-humeral rhythm by Lateral Scapular Slide Test (LSST), and performance by closed kinetic chain test. The training group performed the exercises for 10 weeks. After the exercises, the post-test was administered. To analyze the data, analysis of co-variance tests and paired t-test at the level of 0.05 were employed.

**Results:**

The research results showed that corrective exercises have a significant effect on abnormalities of forward head, forward shoulder, kyphosis, scapula-humeral rhythm and performance.

**Conclusions:**

Corrective exercises can be effective in reducing shoulder girdle and spine abnormalities and improving scapula- humeral rhythm and performance of volleyball players.

## Introduction

Athletes can be identified with special body types and postures; in fact, one of the distinguishing characteristics of athletes is their posture, which prominently differentiates them from others [1]. To achieve any progress, athletes must undertake long-term training programs [2]. Depending on the type of sports activity, each athlete may be susceptible to a certain type of small postural abnormalities or deviations for which exercise is suitable [2, 3], and these postural deviations may lead to a wide range of disorders [4]. The shoulder joint is one of the most important joints involved in overhead sports such as volleyball, tennis, handball, baseball, swimming and badminton. Athletes of these fields are at high risk of shoulder injuries due to repetitive movements and throwing from above the head at an angle over 90 degrees, and also because of the high force and load that is applied to it [5]. Shoulder impingement syndrome is also most common among these athletes [6, 7], which is caused by special patterns of muscle imbalance, including weakness of the middle and lower trapezius, serratus anterior, infraspinatus, deltoid, and shortness of the upper trapezius, pectoral and levator scapulae muscles. This pattern of muscle imbalance was proposed by Janda as the upper cross syndrome, which is mostly associated with abnormalities of forward head, forward shoulder, deviated scapula, and increased thoracic kyphosis [8]. As a supporting structure, the spine provides a framework for body movements and plays an important role in maintaining body posture against the effects of gravity. The soft tissues related to it have a crucial role in support and mobility [9] and their imbalance may gradually lead to height imbalance [10].

Improper posture or deviation from the optimal posture would cause abnormal pressure on the body and lead to deviations in posture and increase the risk of injury in the long term [11], which is also common among volleyball players, runners and firefighters [12–14]. It is stated that if the body is in an undesirable position for a long time, some muscles will be stretched and some will be shortened [15], and the person would gradually adapt to this situation. This adaptation is such that there would be shortness and stiffness in muscles of one side, and weakness and stretching in muscles of the opposite side [16]. In other words, physical activities cause the body to adapt to the required state of that activity. As a result, most athletes may adopt certain physical positions in their relevant fields, like spikes in volleyball, excessive use of the hands over the head and in front of the body and repetition of these movements, causes muscles of the movement group of the shoulder girdle including pectoralis major, upper trapezius, levator scapulae and upper deltoid to shorten; moreover, group of the stabilizing muscles including rhomboids, serratus anterior, posterior deltoid, supraspinatus, and teres minor are prone to weakness and stretching. As a result, the individual would suffer from kyphosis deformity, which causes the forward shoulder deformity as a compensation [17]. Forward head deformity, shoulder and spine changes are among the most common deformities in athletes with overhead movement activities. Plunkett Castilla (2015) [18] reported the prevalence of 85.7% forward head and 42.9% forward shoulders in volleyball and softball athletes. Therefore, it is very important to correct these abnormalities and prevent their secondary effects such as pain and movement limitation. Furthermore, Proper scapular kinematics are very important for optimal shoulder joint function, such as performing repetitive overhead motions [19]. In general, frequent use of the dominant arm would lead to ligament laxity and functional impairment due to joint and muscle contraction [20]. This disorder may lead to inefficient energy transfer and put more pressure on tissues around the shoulder [21], so the kinematics of the scapular movement and scapula-humeral rhythm will change [22]. Voight et al., [23] stated that if the scapula cannot play a stabilizing role, function of the shoulder joint becomes inefficient and performance of the neuromuscular system diminishes, and as a result, the shoulder joint would be damaged. Warner [24] stated that a rehabilitation protocol based on kinetic chain can gradually restore dynamic stability of the scapula by strengthening the stabilizer muscles of the scapula, thus improving muscle activity and muscle balance.

The upper cross syndrome is corrected through various methods, which include manual treatments, postural re-education, use of adhesive tapes and orthotics, and therapeutic exercises. The use of therapeutic or corrective exercises is one of the common methods for correcting abnormalities of the forward head, forward shoulder and increased thoracic kyphosis [8]. Stretching and strength exercises are aimed to correct the posture and also reduce the pain caused by correcting the posture [25]. The objective of every program is to correct posture, restore muscle balance, and normalize range of motion of the joint [4]. Therefore, the purpose of this research is to present a corrective exercise program that emphasizes stretching shortened muscles and strengthening stretched muscles, the effect of such exercises on the degree of Kyphosis, the degrees of forward head and forward shoulder, scapula-humeral rhythm, and performance in patients with upper crossed syndrome. Lynch et al. (2010) investigated the effect of training interventions for 8 weeks and 3 days a week on forward head and shoulder deformity in swimmers aged 17 to 23 years. The results showed that after 8 weeks, the forward head and shoulder angle decreased significantly [3]. Thakur et al. (2014) also concluded that stretching of the scalene muscle and posterior structures of the shoulder, and stabilizing and strengthening the scapular retractors and deep flexor muscles of the neck, reduces the degree of forward head angle in overhead athletes [26]. Also Obayashi et al. (2012) in their research, in order to correct the abnormality of increased thoracic kyphosis, used stretching and strengthening exercises for the thoracic muscles and the posterior muscles of the trunk respectively As a result, the thoracic kyphosis angle decreased after completing the exercise program [27].

Previous studies have typically focused on prescribing stretching exercises for anterior upper trunk muscles and strengthening exercises for extensor muscles, based on Kendall’s theory [16]. However, these studies have often overlooked other hyper-kyphosis-related abnormalities such as forward head posture and forward shoulder, which goes against Vladimir Janda’s theory of interconnected body parts. As a result, the effectiveness of corrective exercises is uncertain due to deficiencies in previous studies and localized exercise program designs. There is a lack of scientific evidence to support these approaches. The corrective exercises utilized in previous research studies did not produce the expected results. These studies, including those conducted by Renno et al. in 2005 [28] ,Vaughn and Brown in 2007 [29], and Bautmans et al. in 2010 [30], only resulted in a decrease of one to three degrees in the kyphosis angle of participants after completing the corrective exercise program. Since adolescent athletes’ spine is still growing and developing until it reaches full maturity like adults’ spine, their skeletal system may go towards asymmetry in an inappropriate position and cause a delay in growth of the soft tissue. Adolescent and young athletes participating in competitive sports are more exposed to the risk of abnormal body posture that may change mechanics of the spine [15]. Based on this and given the contradictions in previous findings and referring to studies conducted by the researcher, no study was found to investigate effects of a course of selected corrective exercises on posture, scapula-humeral rhythm and performance of adolescent volleyball players with upper cross syndrome in a comprehensive and simultaneous manner. This caused the present study to be conducted with the aim of investigating this issue.

## Methods

The present study applied quasi-experimental method with a pre-test post-test design. The statistical population of this research included all athletes with at least 5 years of regular sports experience in volleyball in age range of 16–18 years in Babol city. Among them, with the help of G-power software, 30 athletes with a forward head angle of more than 46 degrees, forward shoulder angle of more than 52 degrees [31], and kyphosis of more than 40 degrees were purposefully selected, and with their consent, randomly assigned in two control and treatment groups (15 in each group). The participants were all healthy (there was no special injury in their shoulder area that would make them go to the doctor and through the course of treatment) and they had no history of back pain or any special injury. Unwillingness to cooperate, injury during exercise or training period, and absence in at least 3 sessions of training were among the exclusion criteria from this study. For the purpose of this study, the participants’ height was measured with a caliper and their weight was measured using a digital scale.

To evaluate kyphosis, a flexible ruler was used with reliability of 0.89–0.92 and validity of 0.91. For this purpose, each subject was asked to stand in front of the evaluator in a natural state without covering the body. Then, the evaluator marked the second and twelfth back vertebrae with a marker. All the measurements were conducted in a relaxed standing position, in such a way that the subjects were asked to put their weight between their legs and look directly to the front. After marking the intended points, the flexible ruler was placed on the spine in such a way that it takes shape of the selected area and there is no empty space between the ruler and the spine. Then, the marked points on the spine were transferred to the ruler. At the end, the ruler was carefully separated from the spine and placed on the target paper, and the curves were drawn on the paper with a pencil and the target points were specified. The distance between two points L and the depth of curvature H were measured by the ruler and then, the kyphosis angle was calculated using the formula Θ = 4arctan 2 H/L [32]. In the present study, forward head and forward shoulder angles was measured using photography method of the body profile view. This method has good reproducibility, such that Ruivo et al., reported intra- and inter-examiner reliability for head forward angle (ICC = 0.87, 0.66) and for forward shoulder angle (ICC = 0.96, 0.78) [33]. To measure angle of the forward head and forward shoulder using this method, first three anatomical signs of ear tragus, right acromion prominence and C7 vertebra spinous appendage were identified and marked with landmarks. Then, the subjects were asked to stand at the designated place next to the wall (at a distance of 23 cm) so that their left arm was towards the wall. Then, the photographic tripod, on which the digital camera was also placed, was put at a distance of 265 cm from the wall and its height was adjusted to the level of the subject’s right shoulder. In such a situation, the subjects were asked to bend forward three times and raise their hands above their head three times, and then stand comfortably and naturally and look at an imaginary point on the opposite wall (eyes in line with the horizon). After a pause of 5 s, the examiner took a picture of the profile view of the body. Finally, the intended photo was transferred to the computer and using AutoCAD software, the angle of the line connecting the tragus and C7 with the vertical line (forward angle of the head) and the angle of the connecting line of C7 and the acromion with the vertical line (angle of the forward shoulder) were measured. The average of the three angles obtained for each abnormality was recorded as the desired angle for forward head and forward shoulder [3, 34, 35].

**Fig. 1:**
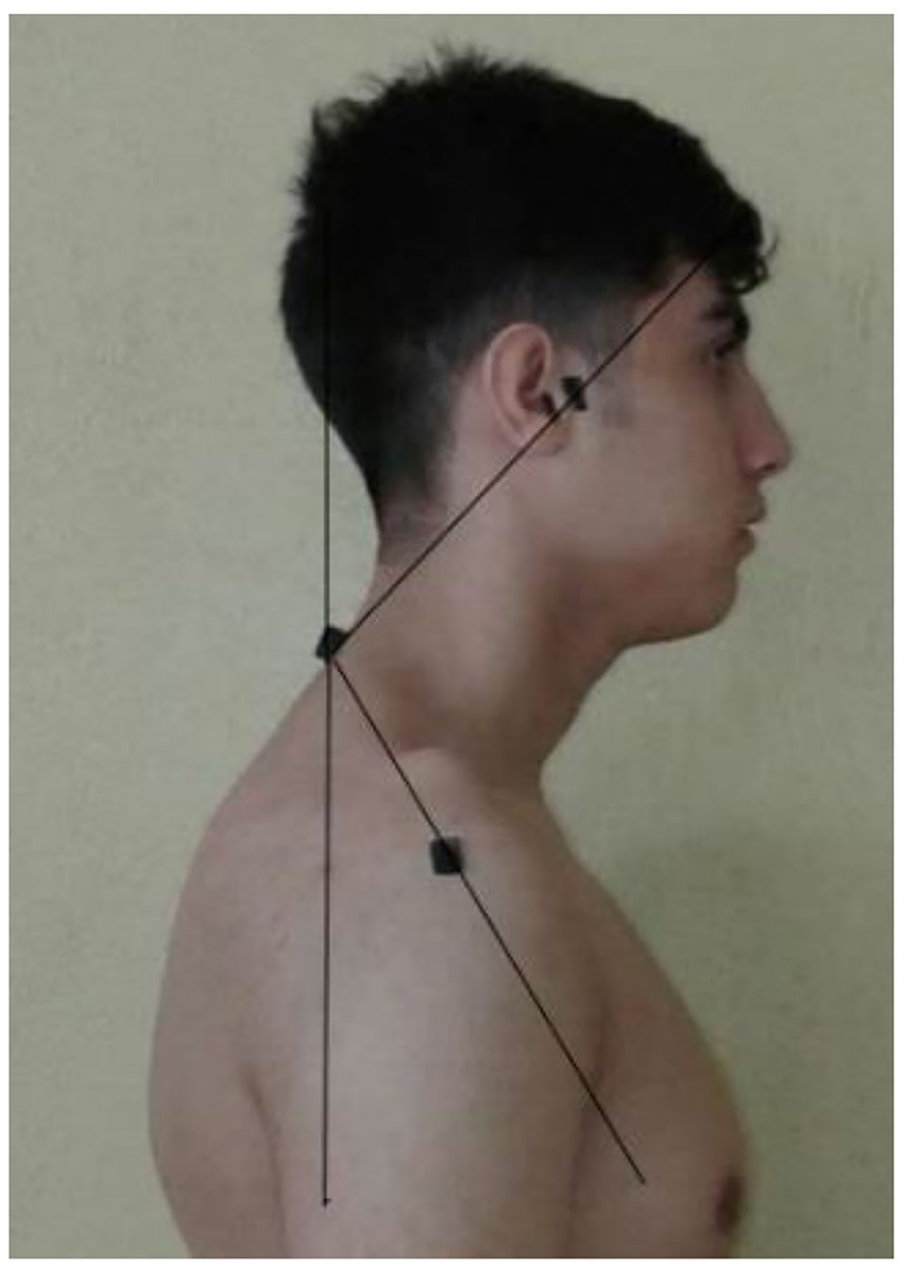
Measurement of forward head/shoulder angles by lateral photographic technique

Lateral scapular slide test (LSST) was employed to evaluate scapula-humeral rhythm. In this test, the subject stood with his back to the examiner. Then, the lower angles of the subjects scapula were marked as a reference point, and the distance between the two lower angles at zero angles (hands were next to the body and the distance of each scapula from the spine was evaluated), 45 degrees (hands were on iliac crest and the distance between the vertebra and the subscapular angle of each side was evaluated) and 90 degrees (the hands were in the position of 90 degrees of abduction and the distance between vertebra and the lower angle of each scapula was evaluated) was measured and recorded using a tape measure (average of 3 measurement load).The current method had a correlation coefficient of 0.92 − 0.91. These measurements were conducted for both left and right scapula. If there is a difference of 1.5 cm or more between the two scapula, the test would be positive [31]. Researchers have considered this test as one of the valid methods for measuring static position of the scapula. intra-rater and inter-rater reliability of this test has been reported as 0.84–0.88 and 0.77–0.85, respectively [22, 36–38]. Also, to evaluate the function of the upper extremity, Closed Kinetic Chain Upper Extremity Stability Test (CKCUEST) was employed. In conducting this test, two 1.5-inch strips were placed in parallel on the ground with a distance of 36 inches. The hands had to be on the strips in Swedish swimming position, then one of the hands was taken off the ground. While maintaining balance on one hand, the guide hand was taken off the ground and got close to the tape that the hand was leaning on it and touched it. Then, they returned to the initial position of Swedish swimming and repeated the movement with the other hand. They repeated the movement for 15 s in a row and alternately for both hands. They performed the movement in 3 sets and rested 45 s between each set (Fig. 1). At the end, the average of three sets or three attempts of the participants was calculated and considered as their score [39].

**Fig. 2:**
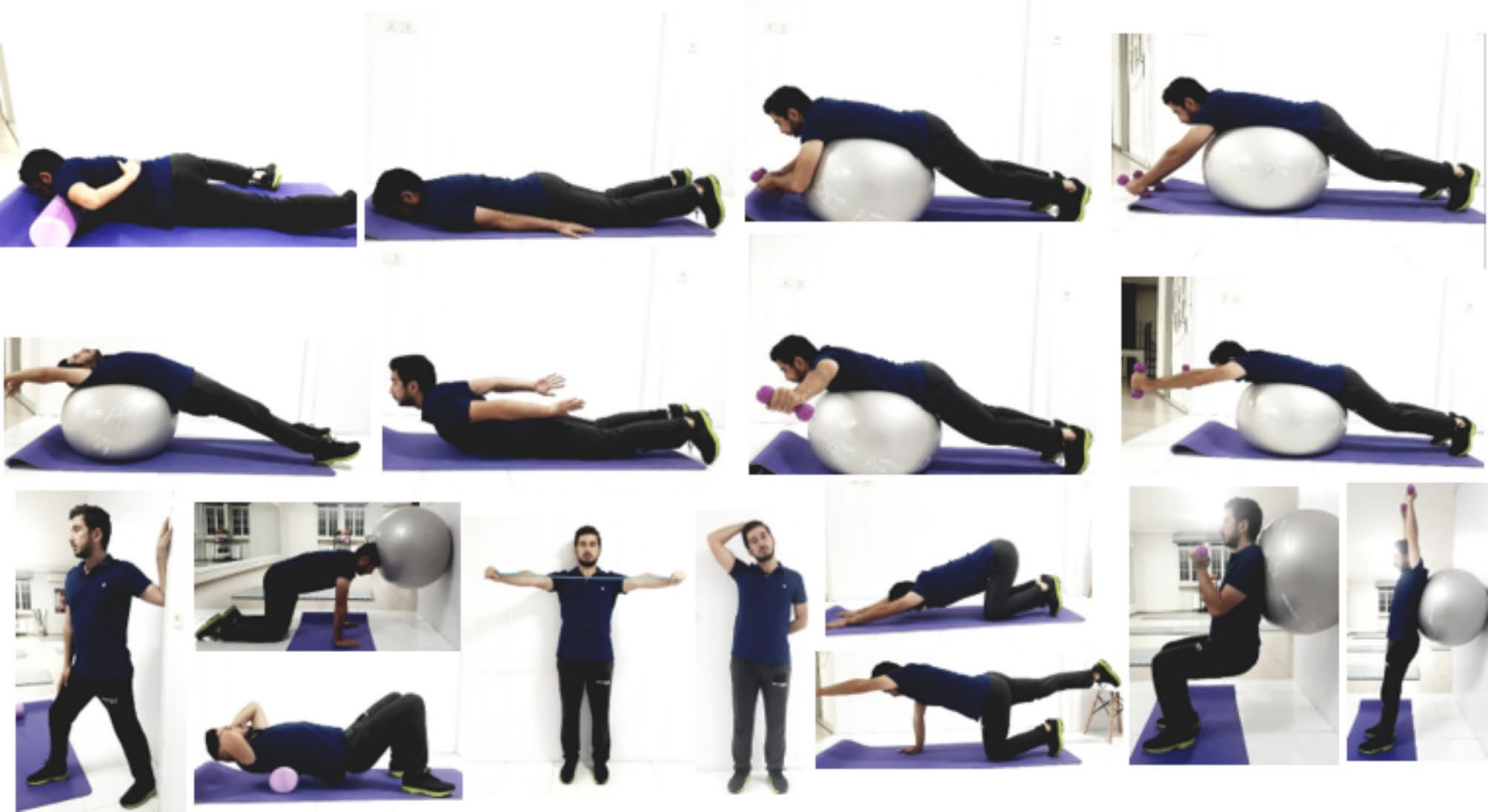
Corrective exercises of the present research

The exercise program was performed for 10 weeks, 3 sessions per week and each session lasted 30–70 min under the supervision of the examiner [40]. The selection of exercises was from easy to difficult, and each session included a neck warm-up (5–10 min), a strengthening and stretching exercise program (60 − 20 min based on gradual progress), and a cool-down (5–10 min). Exercises were performed in a stationary state and their intensity was adjusted for the subjects based on previous findings and their tolerance threshold. Thirteen exercises were provided for the participants in sequence during the training sessions, including: first, releasing muscles of anterior part of the trunk while lying on stomach (prone position) for kyphosis complication; second, releasing the sternocleidomastoid muscle, angular and upper trapezius muscles for the forward head complication; third, stretching muscles of the anterior part of the trunk and shoulder next to the wall; fourth, stretching muscles of the anterior part of the trunk on a Swiss ball; fifth, stretching the sternocleidomastoid muscle, angular and upper trapezius muscles; sixth, stretching the anterior muscles of the trunk and shoulders in quadruped position; seventh, strengthening exercises of the neck area by a Swiss-ball; eighth, Scapular retraction with bandaging and chin tuck; ninth, cobra pose on the ground; tenth, Scaption in prone (face-down) position; eleventh, strengthening back muscles on the ball; twelfth, raising the opposite arm and leg simultaneously in quadruped position on the floor; sixteenth, squat with the ball and overhead press with dumbbells. The training load is brought in the Table below (Fig. 2) (authors Inform consent was obtained from the participant to publish the image). In the first to seventh exercises, the amount of maintaining the movement increased from 10 s to 12 and then 15 s, and in the eighth one, a more resistant thera band was used to increase intensity of the exercise (Table 1).

**Table 1:** Exercise program

	The first exercise	The second exercise	The third exercise	The fourth exercise	The fifth exercise	The sixth exercise	The seventh exercise	The eighth exercise	The Ninth exercise	The tenth exercise	The Eleventh exercise	The twelfth exercise	The thirteenth exercise
1th to 3th week (One minute rest time)	1*8	1*8	1*8	1*8	1*8	1*8	1*8	1*8	3*8	3*8	3*8	3*8	3*8
4th to 6th week (30 s rest time)	1*12	1*12	1*12	1*12	1*12	1*12	1*12	1*12	3*12	3*12	3*12	3*12	3*12
7th to 10th week (30 s rest time)	1*15	1*15	1*15	1*15	1*15	1*15	1*15	1*15	3*15	3*15	3*15	3*15	3*15

(Big numbers: repeat/small numbers: set)

Rest between sets: 45 s

Rest at the end of the set: 90 s

To analyze the collected data, descriptive and inferential statistics methods were applied. Shapiro-Wilk test was employed to check normality of data distribution. In order to compare the average of variables (Forward head, Forward shoulder, Kyphosis,0 degree rhythm, 45 degrees rhythm, 90 degree rhythm and Closed kinetic chain test) between- and within groups, analysis of covariance and t-correlated tests were used at the significance level of 0.05. All statistical operations were performed by SPSS software version 24.

## Results

Mean and standard deviation of the participants’ personal characteristics, including age, height, weight, body mass index, and sports history are displayed in Table 2.

**Table 2:** Demographic characteristics of the two groups

Variable	Control group (15 people)	Training group (15 people)	T	The significance level
	Standard deviation ± mean	Standard deviation ± mean		
Age (years)	16/80 ± 0/77	16/46 ± 0/63	1/28	0/20
Height (meters)	1/75 ± 0/05	1/75 ± 0/03	-0/12	0/89
Weight (kg)	71/86 ± 4/79	74/46 ± 4/10	-1/59	0/12
Body mass index (kg/m^2^)	23/37 ± 1/10	24/19 ± 1/62	-1/61	0/11
Sports history (years)	8/82 ± 1/25	8/11 ± 1/32	1/57	0/15

Results of the independent t-test comparing the individual characteristics of the subjects in two groups proved no significant difference between the groups.

Given the normality of the data, approved by the Shapiro-Wilk test, analysis of covariance and correlated t-test were used to compare between- and within groups. Results of the correlated t- test are brought in Table 3.

**Table 3:** Correlated t-test results for intra-group comparison of abnormalities, scapula humeral rhythm and performance

Group	Control group				Experimental group			
	Pre-test	post-test	T	P	Pre-test	post-test	T	P
Forward head (degrees)	49/99 ± 2/01	50/04 ± 2/00	-0/44	0/66	50/38 ± 1/50	46/32 ± 2/75	7/04	0/001**
Forward shoulder (degrees)	55/61 ± 1/44	55/55 ± 1/42	0/65	0/52	54/96 ± 0/95	51/97 ± 2/41	5/72	0/001**
Kyphosis (degree)	46/61 ± 2/69	45/87 ± 2/28	1/01	0/32	47/28 ± 1/79	43/86 ± 1/63	5/26	0/001**
0 degree rhythm	1/37 ± 0/25	1/33 ± 0/21	1/48	0/15	1/46 ± 0/56	1/20 ± 0/56	2/64	0/001**
45 degrees rhythm	1/56 ± 0/26	1/52 ± 0/24	1/46	0/16	1/80 ± 0/38	1/57 ± 0/53	2/95	0/01**
90 degree rhythm	2/01 ± 0/40	1/98 ± 0/47	0/85	0/40	2/32 ± 0/52	1/69 ± 0/76	3/98	0/001**
Closed kinetic chain test	31/20 ± 2/78	32/33 ± 3/06	-3/01	0/009	31/26 ± 2/25	35/20 ± 2/24	-14/75	0/001**

Significance at the level of 0.01**

Results of the correlated t-test suggest significant impact of corrective exercises on the variables forward head (p = 0.001), forward shoulder (p = 0.001) and kyphosis (p = 0.001), scapula-humeral motor rhythm (p = 0.01), and upper extremity performance (p = 0.001). However, in control group, no significant difference was found between pre-test and post-test. Results of the analysis of covariance test for comparing between groups are presented in Table 4.

**Table 4:** The results of covariance analysis to investigate the effect of independent and predictor variables on anomalies, scapula humeral rhythm and performance

Variable	Test stage	Group	Mean difference	F	df	P	Eta squared
Forward head	post-test	Control group	125/13	47/11	1	0/001**	0/63
	post-test	Experimental group					
Forward shoulder	post-test	Control group	57/29	26/58	1	0/001**	0/49
	post-test	Experimental group					
Kyphosis	post-test	Control group	33/17	8/52	1	0/007**	0/61
	post-test	Experimental group					
0 degree rhythm	post-test	Control group	0/31	4/36	1	0/04*	0/13
	post-test	Experimental group					
45 degrees rhythm	post-test	Control group	0/25	4/70	1	0/03*	0/14
	post-test	Experimental group					
90 degree rhythm	post-test	Control group	2/34	11/30	1	0/002**	0/29
	post-test	Experimental group					
Closed kinetic chain test	post-test	Control group	58/96	36/20	1	0/001**	0/57
	post-test	Experimental group					

* Adjusted based on pre-test values

Significance at the level of 0.001 **

Results of the analysis of covariance test demonstrate that after controlling effect of the pre-test, in results of the variables forward head (p < 0.05), forward shoulder (p = 0.001), kyphosis (p = 0.001), significant difference were found in scapula-humeral motor rhythm (p < 0.05) and upper extremity performance (p = 0.001) in post-test between the two control and treatment groups, such that the amount of these variables is significantly improved in treatment group compared to the other one.

## Discussion

The research results showed that corrective exercises have a significant effect on abnormalities of forward head (p = 0.001), forward shoulder (p = 0.001), and kyphosis (p = 0.007). In addition, scapula-humeral rhythm and performance also showed a significant improvement in treatment group compared to the control one.

In terms of the effect of the exercise program on intended variables, the type of exercises being applied in the program can be considered effective in obtaining results. For example, in the current study, chin tuck was one of the main exercises of the training program to reduce the forward head. Performing such exercises causes the length of the upper shortened muscles at the back of the neck and strength of the muscles in the front part of the neck to increase, which probably leads to the correction of the forward head abnormality by creating a balance between the above muscle groups in terms of length and muscle tension. It seems that in the case of lack of exercises and due to instability in the middle areas of cervical vertebrae and existence of some wrong habits, there is a chance for this posture to return again [41]. In addition, results of the current study proved that performing corrective exercises probably activated muscles of the cervical spine against the stress caused by wrong behaviors and habits, which ultimately leads to the stability of the effects of corrective exercises, such that control of neck movement is improved [42].

In the program of corrective exercises used in present study, a large number of movements and neck flexor muscles, thus, reinforcing their strength and endurance were addressed. Strengthening these muscles may improve the ability to maintain neck posture. Moreover, reduction of head protrusion observed in experimental group may be due to the improvement in endurance of these muscles that occurred during ten weeks of corrective exercises. Also, the program of corrective exercises included stretching and flexibility exercises that focused on stretching the shortened muscles in this complication, i.e., the upper trapezius, levator scapula, and sternocleidomastoid, as well as the pectoralis minor and major muscles [43], which might be helpful in reducing forward shoulder problem and kyphosis. It has been stated in researches that in forward head position, shortening of levator scapula muscle may change its length and tension during the upper rotation of the scapula, and results of the studies also confirm this point. Also, the theory that change in head posture is associated with the change in activity of the scapular muscles is also confirmed [44]. Consequently, strong relationship between the shoulder complex and vertebral column may be another reason for reducing the forward head angle. Therefore, result of the present study suggests that the corrective exercises probably targeted the tissues that improved the posture of forward head and forward shoulder [45]. Grandel et al. (2002) [46] investigated the effect of yoga exercises on improvement of kyphosis and concluded that strength exercises along with flexibility exercises improve kyphosis. Also, Yang et al. (2004) [47] in a study on 58 workers, found increasing the range of trunk movement extension to be an effective factor in reducing kyphosis, hence, reducing back pain. It seems that stretching exercises in anterior part of the trunk as well as strength exercises of trunk extensor muscles, like abduction of the arms in forward bent position can be effective in correcting the kyphosis abnormality. Accordingly, it seems that the use of a corrective program can be effective in reducing these angles, which are related to the sports field of these athletes and working hands overhead, and in this way, the motor performance of these athletes can be improved.

In relation to how the proposed exercises of the current study affect the scapula-humeral rhythm and performance, it can be said that the development of muscle strength causes the scapula-humeral rhythm to improve. The serratus anterior and trapezius are the main muscles that optimize scapular position and scapular rhythm, which, as a result, alleviate pain and enhances performance [48, 49]. However, if the stabilizing muscles of the scapula are weakened or the shoulder performance is impaired, the normal position and kinematics of the scapula would change [23]. Samir et al., [50] stated that muscle strengthening is a potential intervention strategy to improve recovery and prevent shoulder dysfunction. On the other hand, some exercises applied in the current study have caused proximal stability in shoulder joint and created a stable support for proper performance of the upper extremity. In general, it seems that the exercises used in the present study have reduced muscle tension in the scapula and arm complex in individuals with upper cross syndrome, through modifying the relationship between the length and tension of the muscles operating on positioning and stability of the scapula. Since the state of movement, stability and performance of the shoulder is dependent and affected by performance of the scapula, the development and improvement of performance is not far off.

Given the effects created by exercises of the present study, it can be said that alignment of the vertebral column affects position of the scapula, both of which affect performance of the shoulder girdle. Basis of the relationship between alignment of the vertebral column, position of the scapula and performance of the shoulder girdle can be related to the existence of multiple muscle connections between the vertebral column, scapula, clavicle and arm bones. The alignment of these bone parts may directly change through the muscular connections between them. The alignment of the bone affects length of the muscles, through which can affect ability of the muscle to create tension [16, 51]. The efficiency of muscle activity depends on proper orientation and alignment of the scapula on ribcage and the length-tension relationship of the scapular stabilizing muscles and arm rotator muscles. According to the researchers, despite the abnormality of kyphosis, a change in curvature of the ribs causes downward rotation, anterior tilt of the scapula bones, and placement of the shoulders in a position ahead of their anatomical position, which may limit shoulder movement [16]. Therefore, use of corrective exercises can be useful to eliminate motor disorders and limitations in this group.

Among the limitations of the current study were selection of the gender and sports field of the subjects (only male volleyball players). Therefore, in future studies, other researchers can investigate such interventions in other sports fields as well as in female samples.

## Conclusion

According to results of the present study, it seems that implementation of a corrective training program with the aim of preventing and correcting the abnormalities of the trunk, shoulder and scapula area of adolescent volleyball players is effective, and ultimately improves their performance and scapula-humeral rhythm. Therefore, given the significance of these disorders in the spine and shoulder girdle, it is recommended that corrective motor specialists and therapists apply the exercise protocol of the present study to improve and correct the aforementioned postural abnormalities as well as scapular-humeral rhythm and motor performance.

## Data Availability

The datasets generated and analyzed during the current study are not publicly available, as individual privacy could be compromised, but are available from the corresponding author on reasonable request.
